# Approximate Bayesian Inference

**DOI:** 10.3390/e22111272

**Published:** 2020-11-10

**Authors:** Pierre Alquier

**Affiliations:** Center for Advanced Intelligence Project (AIP), RIKEN, Tokyo 103-0027, Japan; pierrealain.alquier@riken.jp

**Keywords:** Bayesian statistics, machine learning, variational approximations, PAC-Bayes, expectation-propagation, Markov chain Monte Carlo, Langevin Monte Carlo, sequential Monte Carlo, Laplace approximations, approximate Bayesian computation, Gibbs posterior

## Abstract

This is the Editorial article summarizing the scope of the Special Issue: Approximate Bayesian Inference.

## 1. Introduction

Extremely popular for statistical inference, Bayesian methods are gaining importance in machine learning and artificial intelligence problems. Indeed, in many applications, it is important for any device not only to predict well, but also to provide a quantification of the uncertainty of the prediction.

The main problem when one is to apply Bayesian statistics is that the computation of the estimators is expensive and sometimes not feasible. Bayesian estimators are based on the posterior distribution on parameters θ given by:

(1) $$\pi \left(\theta |x\right)=\frac{L(\theta ;x)\pi (\theta )}{\int L(\theta ;x)\pi (d\theta )}$$

where π is the prior, *x* the observations, and L(θ;x) the likelihood function. For example, the computation of the posterior mean ∫θπ(dθ|x) requires a difficult evaluation of the integrals. Thanks to the development of computational power, Bayesian estimation became feasible in the 1980s and the 1990s through Markov Chain Monte Carlo (MCMC) methods, such as the Metropolis–Hastings algorithm [1] and the Gibbs sampler [2,3]. These algorithms target the exact posterior distribution. They proved to be useful in many contexts and are still an active area of research. The performances and applicability of MCMC were improved by variants such as the Hamiltonian MCMC [4,5], adaptive MCMC [6,7,8], etc. We refer the reader to the review [9], the books [10,11,12], and Part III in [13] for detailed introductions to MCMC. The surveys [14,15] provide an overview on more recent advances. The asymptotic theory of Markov chains, ensuring the consistency of these algorithms, was covered in the monographs [16,17]. A few non-asymptotic results are also available [18].

Sequential Monte Carlo emerged in the 1990s as a way to update sequentially (that is, for each new data) samples from the posterior in hidden state models. They allow thus the computation of a Bayesian version of filters (such as the Kalman filter [19]). For this reason, they are also referred to as “particle filters”. We refer the reader to [20] for the state-of-the-art of the early years and to the recent books [21,22] for pedagogical introductions and an overview of the most recent progress.

However, many modern models in statistics are simply too complex to use such methodologies. In machine learning, the volume of the data used in practice makes MCMC too slow to be used: first, each iteration of the algorithm requires accessing all the data, then the number of iterations required to reach convergence explodes when the dimension is large. In these cases, it seems that targeting the exact posterior is no longer a realistic objective. This motivated the development of many new methodologies, where the target is no longer the exact posterior, but simply a part of the information contained in it, or an approximation.

Before a short overview of these approximations techniques, let us mention two important examples where approximations were an essential ingredient in the application of Bayesian methods. In 2006, Netflix released a dataset containing movie ratings by its users and challenged the machine learning community to improve on its own predictions for movies that were not rated [23]. Many algorithms were proposed, including methods based on matrix factorization. Bayesian matrix factorization is computationally intensive. The first success at scaling Bayesian methods to the Netflix dataset was based on a mean-field variational approximation of the posterior by [24]. Such approximations will be discussed below.

In computer vision problems, the best performances are reached by deep neural networks [25]. Bayesian neural networks became a popular research direction. A new field of Bayesian deep learning has emerged that relies on approximate Bayesian inference to provide uncertainty estimates for neural networks without increasing the computation cost too much [26,27,28,29]. In particular, References [28,29] scaled these algorithms to the size of benchmark datasets such as CIFAR-10 and ImageNet.

## 2. Approximation in the Modelization

In many practical situations, the statistician is not interested in building a complete model describing the data, but simply in learning some aspects of it. One can think for example of a classification problem where one does not want to learn the full distribution of the data, but only a good classifier. A natural idea is to replace π(θ|x) in (1) by:

(2) $$\tilde{\pi }\left(\theta |x\right)=\frac{\exp\left[-\ell (x;\theta )\right]\pi \left(\theta \right)}{\int \exp\left[-\ell (x;\theta )\right]\pi \left(d\theta \right)}$$

where ℓ(x;θ) is a Taylor loss function—for example, the classification error. When ℓ(x;θ)= −logL(θ;x), we recover (1) as a special case. When ℓ(x;θ)=−αlogL(θ;x) for some α≠1, we obtain tempered posteriors, which appeared for various computational and theoretical reasons in the statistical literature; see [30,31,32,33,34], respectively. The use of the general form (2) was advocated to the statistical community by [35].

It appears that this idea was already popular in the machine learning theory community, where distributions like $\tilde{\pi }\left(\theta |x\right)$ are often referred to as Gibbs posteriors or aggregation rules. The PAC-Bayesian theory was developed to provide upper bounds on the prediction risk of such distributions [36,37,38]. We refer the reader to nice tutorials on PAC-Bayes bounds [39,40]. References [41,42,43] emphasized the connection to information theory. Note that the dropout technique used in deep learning to improve the performances of neural networks [44] was studied with PAC-Bayes bounds in [40]; see also [26]. Many publications in the past few years indeed confirmed that PAC-Bayes bounds are very well suited to analyze the performances of deep learning [45,46,47,48,49,50,51]. See [52] for a recent survey on PAC-Bayes bounds.

Such distributions were also well known in game theory and in prediction with expert advice since the 1990s [53,54]. We refer to the book [55], the recent work [56], and to connected problems such as bandits [57,58].

Finally, many aggregation procedures studied in high-dimensional statistics can also be written under the form of (2); see [59,60,61,62,63,64] with various regression or classification losses. References [65] used a Gibbs posterior based on the quantile loss to estimate a VaR (Value at Risk, a measure of risk in finance).

## 3. Approximation in the Computations

Many works have been done in the past few years to compute estimators based on π(θ|x) or $\tilde{\pi }\left(\theta |x\right)$ in complex problems, or with very large datasets. Very often, this is at the cost of targeting an approximation rather than the exact posterior. It is then important to analyze the accuracy of the approximation.

The nature and accuracy of these approximations are extremely different from one algorithm to the other, and some of them are not well understood theoretically. Below, we group these algorithms into three groups. In Section 3.1, we present methods that still essentially rely on simulations. In Section 3.2, we present asymptotic approximations. Finally, in Section 3.3, we present optimization based methods (this grouping is for the ease of exposition and is of course a little crude; each subsection mentions methods that have little to do with each other).

### 3.1. Non-Exact Monte Carlo Methods

Monte Carlo methods based on Langevin diffusions were introduced in physics in the 1970s [66]. Let ${\left(U_{t}\right)}_{t\geq 0}$ be a diffusion process given by the stochastic differential equation:

$$dU_{t}=\nabla \log\pi \left(U_{t}|x\right)dt+\sqrt{2}dW_{t},$$

where ${\left(W_{t}\right)}_{t\geq 0}$ is a standard Brownian motion. It turns out that the invariant distribution of $\left(U_{t}\right)$ is π(·|x). A discretization scheme with step h>0 leads to the Markov chain ${\tilde{U}}_{n+1}$ $={\tilde{U}}_{n}+h\nabla \log\pi \left(U_{n}|x\right)+\sqrt{2h}\xi_{n}$, where the $\left(\xi_{n}\right)$ are i.i.d standard Gaussian variables. However, it is important to note that $\left(U_{n}\right)$ does not admit π(·|x) as an invariant distribution. Thus, the Langevin Monte Carlo method is not exact (it would become exact with h→0). Reference [67] proposed a correction of this method based on the Metropolis–Hastings algorithm, which leads to an exact algorithm, known as the MALA (the Monte Carlo Adjusted Langevin Algorithm). The Langevin Monte Carlo and MALA became popular in statistics and machine learning following [68]. This paper studies the asymptotic properties of both algorithms. Surprisingly, the exact method does not necessarily enjoy the best asymptotic guarantees. More recently, in the case where $\log\pi (U_{n}|x)$ is concave, non-asymptotic guarantees where proven for Langevin Monte Carlo with a running time that depends only polynomially on the dimension of the parameter θ; see [69,70,71,72,73,74]. Such results are usually not available for exact MCMC methods.

The implementation of the classical Metropolis–Hastings algorithm requires being able to compute the ratio $L\left(\theta ;x\right)/L\left(\theta^{\prime }|x\right)$ for any $\theta ,\theta^{\prime }$. In some models with complex likelihoods, or with intractable normalization constants, this is not possible. This led to a new direction, that is approximations of this likelihood ratio. A surprising and beautiful fact is that, if each likelihood is computed by an unbiased Monte Carlo estimator, the algorithm remains exact: this was studied under the name pseudo-marginal MCMC in [75]. Still, it sometimes requires much work to get unbiased estimates [76,77], when possible at all. Some authors proposed more general approximations of the likelihood ratio, leading to non-exact algorithms. References [78,79,80,81] proposed estimators based on subsampling when the data *x* are too large. Reference [82] proposed an estimator of the likelihood ratio when the likelihood has intractable constants, as in the exponential random graph model, and proved that, even if the resulting MCMC is inexact, it remains asymptotically close to the exact chain. A further theory was developed in [83,84,85]. More on MCMC for big data can be found in [86].

Finally, the ABC (Approximate Bayesian Computation) algorithm was proposed in population genetics for models where the likelihood is far too complex to be computed, but where it is relatively easy to sample from it [87,88]. It became extremely popular in some applications; we refer the reader to the survey [89], to Section 3 in [15], and more recently, to the book [90]. Some theoretical results were proven in [91]; we also refer the reader to [92,93,94] for some recent advances.

### 3.2. Asymptotic Approximations

Laplace’s method provides a Gaussian approximation of the posterior centered on the Maximum Likelihood Estimator (MLE) and whose covariance matrix is the inverse of the Fisher information. This approximation can be theoretically justified in parametric models under appropriate regularity conditions thanks to the Bernstein–von Mises theorem. We refer the reader to Chapter 13 in [95] for a complete statement of this result. Integrated Nested Laplace Approximations (INLA) indeed became very popular in Gaussian latent models to compute approximations of the posterior marginals [96].

The extension of the Bernstein–von Mises theorem to nonparametric or semiparametric models is a quite technical and important research direction; see for example [97,98,99,100,101] and Chapter 10 in the monograph [102]. It is important to keep in mind that even in parametric models, when the assumptions of the theorem are not met, Laplace approximation can be wrong. The asymptotic of the posterior in such models was studied in detail in [103].

### 3.3. Approximations via Optimization

A huge number of methods are based on the idea of using optimization algorithms to find the best approximation of π(·|x), or $\tilde{\pi }\left(\cdot |x\right)$, in a set of probability distributions Q fixed by the statistician. The difference between the various methods is in the choice of the criterion used to define the “best” approximation. The set Q can be parametric (e.g., Gaussian distributions, inspired by Laplace’s method) or not, the choice being prescribed by the feasibility of the optimization problem.

Variational approximations are based on the Kullback–Leibler divergence KL:

(3) $$\begin{array}{ll} \hat{\pi }\left(\theta |x\right) & =\underset{q\in Q}{\mathrm{argmin}}KL\left(q||\pi \left(\cdot |x\right)\right) \end{array}$$

(4) $$\begin{array}{c} =\underset{q\in Q}{\mathrm{argmin}}\left\{E_{\theta ∼q}\left[-\log L\left(\theta ;x\right)\right]+KL\left(q||\pi \right)\right\}, \end{array}$$

where we remind that KL(q||p)=∫log(dq/dp)dp when *q* is absolutely continuous with respect to *p*, and KL(q||p)=+∞ otherwise. We refer the reader to the seminal papers [104,105], to the tutorial [106], and to the recent review of the huge literature on variational approximations [107]. Note that the approximation used in [108] in the early days of neural networks can also be interpreted as a variational approximation. Besides the aforementioned applications to recommender systems and to deep learning, variational inference was successfully used in network data analysis [109], economics and econometrics [110,111,112,113], finance [114], natural language processing [115], and video processing [116], among others. A huge range of optimization algorithm were used, from the coordinate-wise optimization in the original publications to message passing [117], the gradient and stochastic gradient algorithm [27,115,118], and the natural gradient [119]. The convexity and smoothness of the minimization problem were discussed in [120]. The scope of these methods was extended to models with intractable likelihood in [121]. Reference [122] pointed out a connection between (4) and PAC-Bayes bounds, which led to the first generalization error bounds for variational inference for some Gibbs posteriors, as in (2). The analysis was extended to various settings, including regular posteriors, as in (1), by [123,124,125,126,127,128,129,130,131]. In particular, Reference [132] proved that variational inference leads to the optimal estimation of some classes of functions with deep learning. Note that even when Q is the set of all Gaussian distributions on the parameter space, the approximation can be very different from the Laplace approximation. Indeed, Reference [129] contains an example of a mixture model where the MLE is not consistent, but Gaussian variational inference is.

The choice of the Kullback–Leibler divergence in (3) and (4) was initially motivated by the tractability of the computational program to which it leads. Recently, many authors questioned that choice and proposed extended definitions of variational inference using other divergences; for a presentation of the most popular divergences in statistics, see the introduction to information geometry [133]. Note that if we replace KL by another divergence, (3) and (4) are in general no longer equivalent, which leads to two possible ways to extend the definition. Reference [134] extended (3) by replacing the KL term by a Rényi divergence, and Reference [135] used the $\chi^{2}$ divergence. However, Reference [136] discussed the computational difficulties induced by these changes, which might outweigh the benefits. Reference [137] discussed other criteria, including the Wasserstein distance, and provided some theoretical guarantees. On the other hand, References [138,139,140,141] proposed to use more general divergences in (3). This can be related to the generalized exponential family of [142] and the PAC-Bayes bounds in [143,144].

The very popular Expectation Propagation algorithm (EP) was introduced by [145]. EP can be interpreted as the minimization of the reverse KL, KL(π(·|x)||q)), instead of (3). This was detailed in [146], where the author also proposed an extension with α-divergences called power EP. Algorithmic issues were discussed in [147] and by [148], who proposed stochastic optimization methods. A first theoretical analysis of EP was proposed in [149]. Let us mention that the textbook [150], which is a generalist introduction to machine learning, contains a full chapter entirely devoted to a pedagogical introduction to variational approximations and EP. The paper [151] focuses on the application of EP to hierarchical models, but also contains a very nice introduction to EP and the conditions ensuring its stability.

Finally, let us mention approximations by discrete distributions, of the form $q=\frac{1}{M}\sum_{i=1}^{M}\delta_{\theta_{i}}$ where $\delta_{x}$ is the Dirac mass at *x*. Note that this is typically the kind of approximation provided by the MCMC and sequential Monte Carlo methods, but in these methods, the $\theta_{i}$ are sampled. It is also possible to try to minimize a distance criterion between *q* and π(·|x). Unfortunately, when π(·|x) is continuous, both KL(π(·|x)||q))=KL(q||π(·|x)))=+∞, so it is not possible to use variational inference or EP in this case. An energy based criterion was proposed in [152]. Reference [153] proposed to use Stein divergences between *q* and π(·|x), and the technique became quite successful [154,155,156]. Another possible research direction is to use the Wasserstein distance [157].

## 4. Scope of This Special Issue

The objective of this Special Issue is to provide the latest advances in approximate Monte Carlo methods and in approximations of the posterior: the design of efficient algorithms, the study of the statistical properties of these algorithms, and challenging applications.

## Abbreviations

The following abbreviations are used in this manuscript:

ABC	Approximate Bayesian Computation
EP	Expectation Propagation
MALA	Monte Carlo Adjusted Langevin Algorithm
MCMC	Markov Chain Monte Carlo
MLE	Maximum Likelihood Estimator
PAC	Probably Approximately Correct
VaR	Value at Risk
